# Isolating sensory artifacts in the suprathreshold TMS-EEG signal over DLPFC

**DOI:** 10.1038/s41598-023-29920-2

**Published:** 2023-04-26

**Authors:** Mohsen Poorganji, Reza Zomorrodi, Colin Hawco, Aron T. Hill, Itay Hadas, Christoph Zrenner, Tarek K. Rajji, Robert Chen, Daphne Voineskos, Daniel M. Blumberger, Zafiris J. Daskalakis

**Affiliations:** 1 Temerty Centre for Therapeutic Brain Intervention, Centre for Addiction and Mental Health, Toronto, ON Canada; 2 Institute of Medical Science, University of Toronto, Toronto, ON Canada; 3 Department of Psychiatry, University of Toronto, Toronto, ON Canada; 4 Cognitive Neuroscience Unit, School of Psychology, Deakin University, Melbourne, VIC Australia; 5 Krembil Research Institute, University Health Network, Toronto, ON Canada; 6 Division of Neurology, Department of Medicine, University of Toronto, Toronto, ON Canada; 7 Department of Psychiatry, School of Medicine, University of California San Diego, 9500 Gilman Drive, La Jolla, CA 92093-0603 USA; 8 Department of Neurology and Stroke, and Hertie Institute for Clinical Brain Research, University of Tübingen, Tübingen, Germany; 9 Toronto Dementia Research Alliance, University of Toronto, Toronto, ON Canada

**Keywords:** Neuroscience, Medical research

## Abstract

Combined transcranial magnetic stimulation and electroencephalography (TMS-EEG) is an effective way to evaluate neurophysiological processes at the level of the cortex. To further characterize the TMS-evoked potential (TEP) generated with TMS-EEG, beyond the motor cortex, we aimed to distinguish between cortical reactivity to TMS versus non-specific somatosensory and auditory co-activations using both single-pulse and paired-pulse protocols at suprathreshold stimulation intensities over the left dorsolateral prefrontal cortex (DLPFC). Fifteen right-handed healthy participants received six blocks of stimulation including single and paired TMS delivered as active-masked (i.e., TMS-EEG with auditory masking and foam spacing), active-unmasked (TMS-EEG without auditory masking and foam spacing) and sham (sham TMS coil). We evaluated cortical excitability following single-pulse TMS, and cortical inhibition following a paired-pulse paradigm (long-interval cortical inhibition (LICI)). Repeated measure ANOVAs revealed significant differences in mean cortical evoked activity (CEA) of active-masked, active-unmasked, and sham conditions for both the single-pulse (F(1.76, 24.63) = 21.88*, p < *0.001, η^2^ = 0.61) and LICI (F(1.68, 23.49) = 10.09*, p < *0.001, η^2^ = 0.42) protocols. Furthermore, global mean field amplitude (GMFA) differed significantly across the three conditions for both single-pulse (F(1.85, 25.89) = 24.68*, p < *0.001, η^2^ = 0.64) and LICI (F(1.8, 25.16) = 14.29*, p < *0.001, η^2^ = 0.5). Finally, only active LICI protocols but not sham stimulation ([active-masked (0.78 ± 0.16, *P < *0.0001)], [active-unmasked (0.83 ± 0.25, *P < *0.01)]) resulted in significant signal inhibition. While previous findings of a significant somatosensory and auditory contribution to the evoked EEG signal are replicated by our study, an artifact attenuated cortical reactivity can reliably be measured in the TMS-EEG signal with suprathreshold stimulation of DLPFC. Artifact attenuation can be accomplished using standard procedures, and even when masked, the level of cortical reactivity is still far above what is produced by sham stimulation. Our study illustrates that TMS-EEG of DLPFC remains a valid investigational tool.

## Introduction

Transcranial magnetic stimulation (TMS) is a valuable non-invasive brain stimulation technique which can be used to both examine, and modulate, cortical activity. TMS combined with electroencephalography (TMS-EEG) can be used to directly perturb the brain and record its response. The field of TMS-EEG is garnering much attention due to its ability to reliably investigate key neurophysiological processes within the cortex^1,2^. There have been many advancements in TMS-EEG as research continues to optimize methods of recording the neural signal in the presence of TMS artifacts. The current standard of TMS-EEG recordings is focused on the appropriate stimulation intensity, precise targeting of the stimulation site, minimizing artifacts associated with somatosensory and auditory co-activation, as well as proper training of technicians, scientists, and clinicians^3,4^.

Although sensory co-activations, which are reflected in the TMS-EEG recording, are genuine brain responses, they can mislead researchers to consider them the result of direct cortical activation by the TMS pulse; therefore since the researchers are not directly interested in these signals, they have been called sensory artifacts^1,5,6^. The presence of auditory and somatosensory artifacts in the TMS-EEG signal has been reported across a number of studies^7–12^. It was shown that applying foam and playing white noise results in differences to TMS-evoked potentials compared to the conventional method of recording TMS-EEG^13^. To avoid the co-activation of auditory pathways and minimize somatosensory co-activation, application of a spacer (air gap^14^ or a layer of foam between the TMS coil and the scalp^13^), as well as playing of white noise to reduce the influence of the auditory coil ‘click’ during the recording is recommended^12,15^. Although many groups have studied the somatosensory and auditory artifacts contained within the TMS-EEG signal following stimulation of the motor cortex, this topic is under-studied for the suprathreshold TMS-EEG over the prefrontal cortex. The question remains whether auditory masking and foam spacing (to minimize the sensory artifacts) would cause different excitatory and inhibitory responses at supra-threshold stimulation intensity and over the prefrontal cortex in comparison with older methods of delivering TMS-EEG. In a recent publication^10^, our group has quantified the contribution of auditory co-activation on the TMS-EEG response over left dorsolateral prefrontal cortex (DLPFC) at suprathreshold levels of stimulation. However, we neither investigated the distinct somatosensory artifact related to stimulation, nor the effect of applying advanced artifact reduction methods (i.e., playing white noise and applying a layer of foam) on TMS-EEG recordings over this region.

The current study was designed to compare the neurophysiological responses to various stimulation conditions at the left DLPFC. It was shown by Rocchi et al.^12^ that suppression of auditory evoked responses can be effectively achieved if masking is done properly. Following the same logic over DLPFC, the conditions in our study included active-masked (TMS with auditory masking and foam spacing), active-unmasked (TMS without auditory masking and foam spacing), and sham (sham coil). This is the first time that the effect of masking the auditory and minimizing the somatosensory (as much as it is possible) co-activation has been explored for both single-pulse and paired-pulse (long-interval cortical inhibition (LICI)) protocols over the prefrontal cortex. LICI is an important neurophysiological measurement of TMS-EEG that is linked to GABAergic neurotransmission and implicated in the pathophysiology and treatment of psychiatric disorders^16,17^. Therefore, we were also interested in investigating whether LICI is affected by the auditory and somatosensory co-activations, or if it reflects the inhibition induced by primary effect of TMS to the neuronal populations. Several lines of research have shown that single-pulse and paired-pulse suprathreshold TMS-EEG of DLPFC can be effectively used as a potential biomarker in psychiatric disorders^16,18–20^. However, the concern regarding the sensory co-activation of this protocol of stimulation has not been addressed previously. We chose a comparatively high TMS intensity in order to ensure that we achieved cortical stimulation in addition to significant sensory co-activation. We hypothesized that single-pulse TMS and the paired-pulse protocol would result in larger response amplitudes in the active-unmasked condition compared to the active-masked (without the same degree of sensory co-activation) and sham (sensory elicitation only—without direct TMS brain responses) conditions. We also hypothesized that there would be a significant difference between the active-masked condition (larger amplitude) and sham. Moreover, we hypothesized that the scalp topographic distribution of cortical evoked activity would be different between each of the three conditions. Finally, we hypothesized that LICI (signal inhibition) would be significant in both active-masked and active-unmasked conditions but not in the sham condition.

## Methods

### Participants

**Figure 1:**
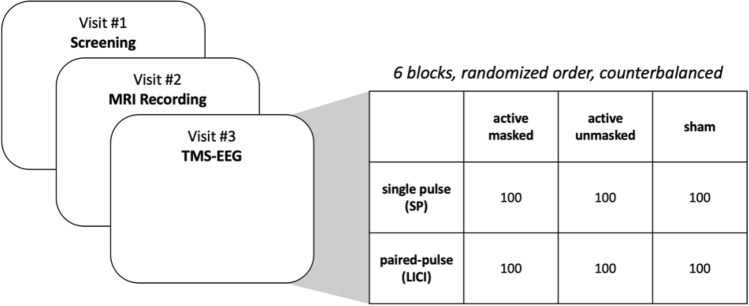
Study design. On the first visit, the participant was screened to meet the inclusion criteria. On the second visit, structural (T1 and T2) magnetic resonance imaging (MRI) scans were performed. On the third visit, the participant received six blocks of stimulation including single-pulse (SP) and long-interval cortical inhibition (LICI) active-masked (with auditory masking and foam spacing), active-unmasked (without auditory masking and foam spacing), and sham.

Fifteen healthy participants (right-handed, aged between 19 and 48 years, 7 female, non-smoker, fluent in English, not meeting the criteria for any DSM-IV Axis I disorder) underwent the three separate testing sessions (outlined in Fig. 1). The protocol of the study was approved by the Centre for Addiction and Mental Health ethics committee. All of the participants provided their informed written consent prior to participating in this study. All of the experiments were performed in accordance with the guidelines and regulations that were approved by the ethics committee. The exclusion criteria consisted of positive drug test (urine screening test), any contradictions to TMS, history of a medical or neurological disorder affecting the central nervous system (CNS), pregnancy, history of seizures, metal implant or dentures, serious or unstable medical condition, and presence of cardiac pacemaker, cochlear implant or any other contraindications to magnetic resonance imaging (MRI). The participants were first screened regarding the inclusion and exclusion criteria. On the second visit, participants underwent T1- and T2-weighted MRI scans of the brain. Subsequently, the participants underwent 6 blocks of TMS-EEG recordings.

### TMS

Active TMS pulses were administered using a 70 mm figure-of-eight coil with a BiStim^2^ TMS stimulator (Magstim Company Ltd., UK). For sham stimulation, a sham coil (70 mm, figure-of-eight) was used with a Magstim Rapid^2^ TMS stimulator (Magstim Company Ltd., UK). At the beginning of the session, resting motor threshold (RMT) was determined while the participant had the EEG cap on. TMS pulses were delivered to left motor cortex while recording motor-evoked potentials (MEPs) from a contralateral hand muscle (abductor pollicis brevis) using surface electromyography (Cambridge Electronic Design Limited, UK). RMT was determined using standard methods as the intensity that resulted in an MEPs with an amplitude exceeding 50 µV in 5 out of 10 consecutive trials^21^. Additionally, the TMS intensity resulting in MEPs with an average peak-to-peak amplitude of 1mv was determined (SI_1mv_) and used as stimulator intensity for both single pulse and LICI protocols in active and sham stimulations^21^. The TMS-EEG blocks were delivered over the left DLPFC. The motor cortex and DLPFC identification and targeting were performed via the Brainsight neuronavigation system (Rogue Research, Montreal, Canada). Based on each participant’s MRI scan, the left DLPFC target was acquired according to Montreal Neurological Institute (MNI) coordinates (x-35; y45; z38)^22^. Each of the 6 blocks of the TMS-EEG session consisted of 100 pulses administered at a frequency of 0.2 Hz and unjittered. The blocks were randomized. The single-pulse protocol consisted of a single suprathreshold TMS pulse per trial, and the LICI protocol comprised 2 suprathreshold stimuli that were separated by 100 ms^23^.

### Electroencephalography (EEG) recording

To record the EEG signal, a 64 channel Ag/AgCl electrode EEG cap (Quick-cap) was used. The cap was connected to a SynAmps2 amplifier and the signal was recorded using Curry software (Compumedics, Version 8). The data were recorded with a sampling rate of 20 kHz. EEG data were recorded in DC mode (i.e., without a high-pass filter) with a 3500 Hz low pass-filter. To ensure high quality EEG recordings, the impedance of the electrodes was first checked and kept below 5 kΩ throughout the recording. Secondly, before starting the recording over the left DLPFC, several test pulses were applied and the response averaged to verify minimal TMS artifact by reducing the facial and cranial muscle twitches if any were noted.

### TMS-EEG paradigms

Each participant received six different blocks of stimulation (Fig. 1). The stimulation blocks consisted of single-pulse and LICI active with foam spacing and auditory masking (active-masked), single-pulse sham and LICI sham, and single-pulse and LICI with neither foam spacing nor auditory masking (active-unmasked). For foam spacing, a layer of foam (Electro-Cap International, Inc, USA) was attached to the center of the coil to minimize the sensory co-activation of the stimulation. To mask the auditory effect, the participant wore in-ear headphones which were further covered by a set of earmuffs. White noise was played for the participants (to suppress the auditory evoked response) based on the maximum volume that was tolerable for them. The masking condition is comparable to the ATTENUATE procedure of masking the sensory co-activation of suprathreshold TMS-EEG of DLPFC, recently introduced by Ross et al.^24^.

### Data pre-processing

Before pre-processing the data, the researcher who pre-processed the data was blinded to each of the stimulation conditions. TMS-EEG data were cleaned using standard procedures, as previously reported (Hadas et al. 2019). Briefly, the pre-processing steps were performed with custom MATLAB (R2018b) scripts using the FieldTrip^25^ and EEGLAB^26^ toolboxes. First, the data were segmented into epochs from -2450 to + 2450 ms around the TMS pulse. Data were cut and linearly interpolated from − 2 to 15 ms around the TMS pulse in order to remove data containing the high amplitude TMS pulse artifact. The next steps consisted of removing the noisy epochs and channels, and downsampling the data from 20,000 to 1000 Hz. Then, channel interpolation (to recreate the previously rejected channels) and baseline correction was applied before running the first round of independent component analysis (ICA–FastICA) to remove the TMS decay artifact. The bandpass zero-phase shift filter (1–100 Hz) with a notch filter of 58–62 Hz was applied. To avoid ripples before and after the TMS pulse, the bandpass filter was only applied after the TMS pulse was removed, the data were trimmed, and the first round of ICA was applied to remove the remaining effects of the TMS pulse. A second round of ICA was then performed to remove any remaining artifacts in the EEG signal.

### TMS-EEG outcome measures

One of the outcome measures of TMS-EEG response is the TMS-evoked potential (TEP)^1,27^. The TEP is a waveform that is both time- and phase-locked to the TMS pulse. It starts at the time of the stimulation and lasts for approximately 300–400 ms^27^. LICI was measured using Eq. (1) (below) where the corrected waveform is the result of subtracting 100 ms-shifted single pulse from the LICI protocol TEP^16,23^. Area under the curve of TEP waveform, either local or global, is shown to reflect the cortical reactivity to TMS^28^. We define cortical evoked activity (CEA) as the area under the curve of the rectified TEP waveform that encapsulates all the peaks and troughs of TEP waveform from the site of the stimulation (F3 electrode, which was approximately located near the site of the stimulation)^16^. The CEA is measured for 2 time windows of CEA _(25–275 ms)_ (between 25 and 275 ms), and CEA _(25–80 ms)_ (25 to 80 ms [to avoid the N100 and encapsulate the early components of P30, N45, and P60]). In addition to the activity recorded from the stimulation site, we investigated the effect of sensory co-activation and masking it on the global TMS-EEG response, recorded across all of the electrodes. To do so, global mean field amplitude was calculated based on Eq. (2)^29,30^. Like CEA, the area under the curve of GMFA is measured for the two time windows of 25 to 80 ms and 25 and 275 ms (GMFA _(25–80 ms)_, and GMFA _(25–275 ms)_). The larger time window (25–275 ms) was chosen to encapsulate the overall TEP time window^27^, and the shorter time window (25–80 ms) was selected to only focus on the early evoked activity which was previously shown to be less affected by sensory co-activation in TMS-EEG of motor cortex^11,12^.

To compare responses in different frequency bands, a 6^th^ order zero-shift Butterworth filter was applied where the frequency ranges were considered as delta (1–3 Hz), theta (4–7 Hz), alpha (8–13 Hz), beta (14–30 Hz), and gamma (31–50 Hz). The frequency decomposition was first performed over the entire epoched data and then the corresponding measures were calculated over the time window of interest.

$$\mathrm{LICI}=\frac{Area ~Under ~the ~Crurve({TEP\_paired~ pulse\_Corrected)}_{(25-275~ms)}}{Area ~Under~ the ~Crurve({TEP\_single~ pulse )}_{(25-275~ms)}} (1)$$

$$\mathrm{Global~ Mean ~Field~ Amplitude}=\sqrt{\frac{{\sum_{i}^{K}({V}_{i}(t)-{V}_{mean}(t))}^{2}}{K}} (2)$$

K = all 64 channels.

The amplitude of the TEP components over the stimulation site were measured by visual exploration of the waveforms to detect the peaks and troughs. The first peak and trough after 25 ms were considered as P30, and N45. The amplitudes of the peak and trough after N45 were chosen as P60 and N100 respectively. The P30 component could not be detected for two of the participants in their active-unmasked block of stimulation. These two values were replaced by the average of P30 amplitude for this condition.

The average of the amplitude of the GMFA waveform (activity across all electrodes) was used for measuring the amplitude of the components in it. The time windows for each component were defined as P30: 25 to 35 ms, N45: 35 to 55 ms, P60: 50 to 70 ms, and N100: 80 to 120 ms^20^. The components from the GMFA waveform will be called P30_(GMFA)_, N45_(GMFA)_, P60_(GMFA)_, and N100_(GMFA)_ throughout this manuscript, and the components detected from the stimulation site will be called without and index (i.e. P30, N45, P60, and N100). The TEP components of P30, N45, P60, and N100 were chosen as the main focus of this manuscript as the neuronal mechanisms behind them are well-studied in pharmaco-TMS studies^31^ and have shown potential to be used as biomarkers in psychiatric disorders^2^.

### Statistical analysis

One-way repeated measure ANOVA was used to compare the various measures of the cortical responses including CEA and GMFA of the six blocks of stimulation, and the partial eta-squared was reported as the effect size. Bonferroni correction was used to correct for multiple comparisons. To investigate the interaction effect of the condition (active-masked, active-unmasked, and sham) and protocol (single pulse, and LICI) of the stimulation on CEA, a two-way repeated measure ANOVA was used. A two-way repeated measure ANOVA was also used to study the interaction effect of masking-condition (active-masked and active-unmasked) and time window of response (25–80 ms and 25–275 ms) on CEA and GMFA. To investigate induced inhibition by the LICI protocol within every condition, the non-parametric Mann–Whitney U-test was used to compare the LICI with no excitatory or inhibitory situation. The effect size is reported as r ($\frac{Z}{\surd {N}_{(Observations)}}$)^32^.

#### T-maps

The T-maps are scalp topographical maps that are composed of the t-values of the student t-tests performed to compare the CEA of active-masked and active-unmasked conditions, over every electrode, for both single pulse and LICI protocols. The t-map analysis was performed for both the response in early time window (25–80 ms), and overall (25–275 ms). To correct for multiple comparison in the t-maps, the false discovery rate (FDR) approach was applied using FDR_BH^33^ function in MATLAB (R2018b). Statistical analyses were performed using MATLAB (R2018b) and SPSS (V25).

## Results

### Cortical evoked activity (CEA)

#### Single-pulse TMS

**Figure 2:**
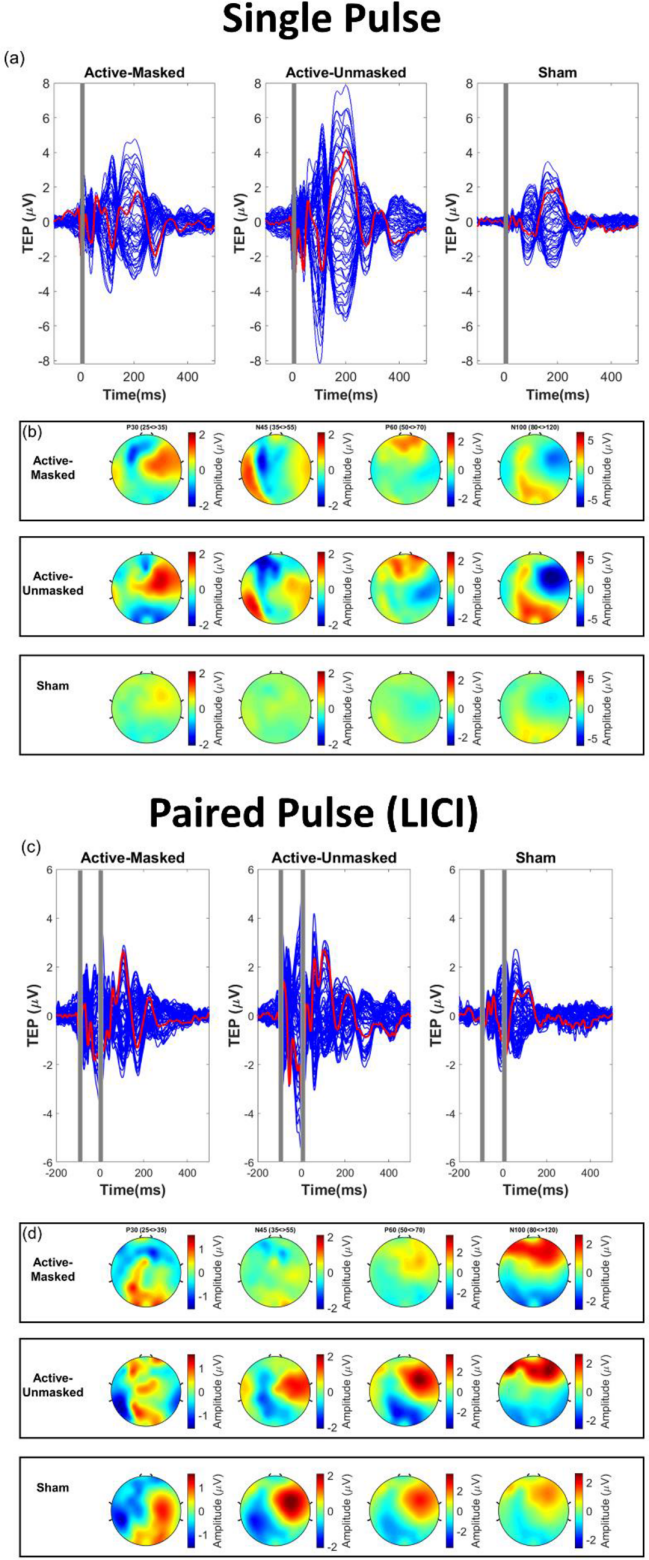
(**a**) Butterfly plot showing the TMS-evoked potential waveforms for each of the three single-pulse stimulation conditions. The red line is the F3 electrode located approximately over the left DLPFC. (**b**) Topoplots of the three different single-pulse stimulation conditions. (**c**) Butterfly plot showing the TMS-evoked potential waveforms for each of the three paired-pulse stimulation conditions. The red line is the F3 electrode located approximately over the left DLPFC. (**d**) Topoplots of the three different paired-pulse stimulation conditions. All plots represent the average across all subjects.

**Figure 3:**
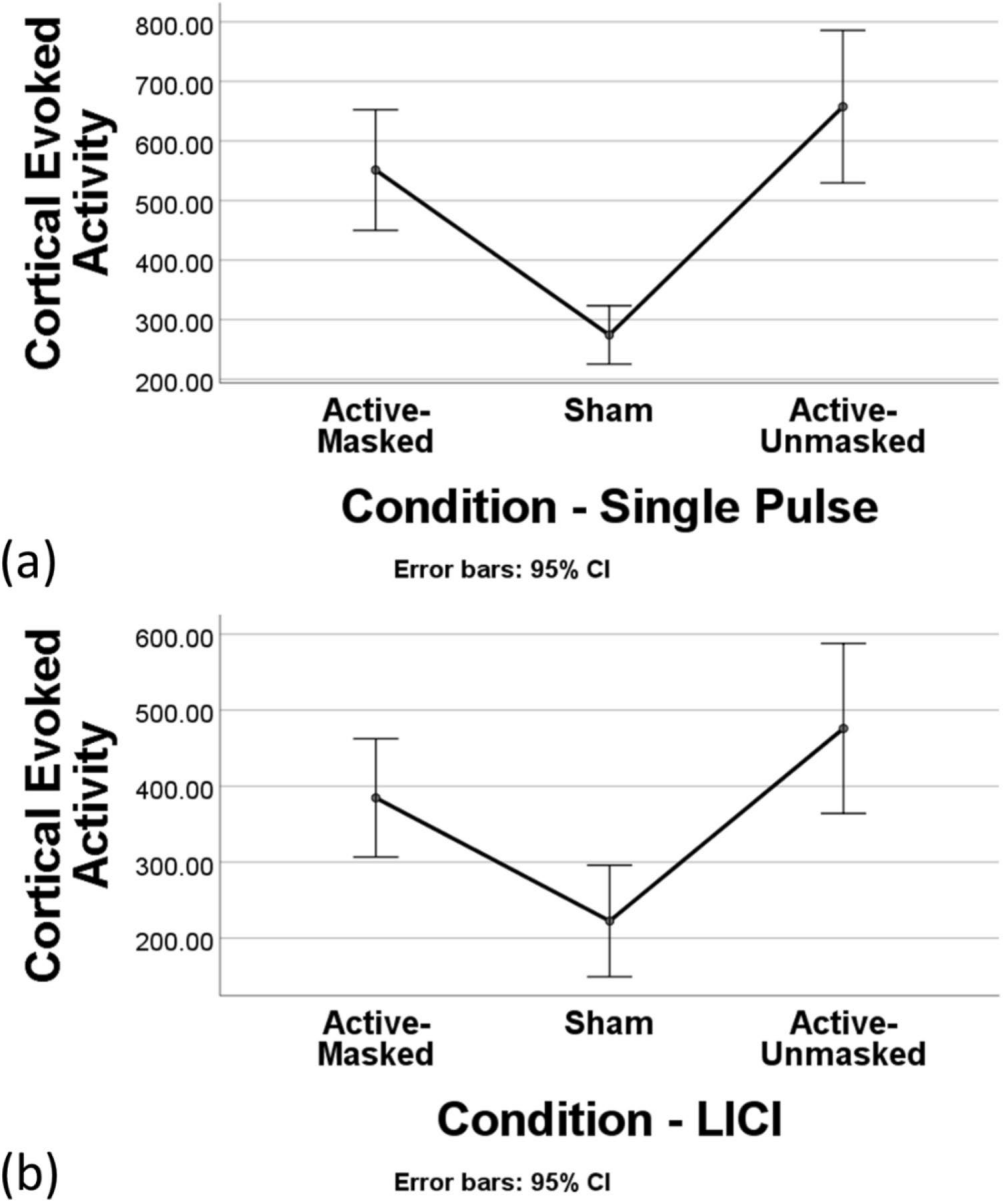
(**a**) Comparison of cortical evoked activity (CEA_(25–275 ms)_) from electrodes F3 of the three single-pulse (SP) stimulation conditions using one-way repeated measure ANOVA. (**b**) Comparison of CEA_(25–275 ms)_ from electrode F3 of the three conditions in paired pulse/ long interval cortical inhibition protocol (PP/ LICI) using one way repeated measure ANOVA.

Significant differences can be seen between the amplitude of the waveforms across three conditions in single-pulse (Fig. 2a,b). A repeated measures ANOVA revealed that CEA _(25–275 ms)_ differed significantly between the three conditions following single pulse TMS (F(2, 28) = 21.88*, p < *0.001, η^2^ = 0.61) (Fig. 3a). Post-hoc analysis (with Bonferroni correction) revealed a significant difference between CEA _(25–275 ms)_ for the active-masked and sham conditions (551.33 ± 182.98 versus 274.49 ± 88.55, *p < *0.001), as well as between the active-unmasked and sham conditions (657.67 ± 231 versus 274.49 ± 88.55, *P < *0.001). However, no significant difference was observed between the CEA _(25–275 ms)_ of active-masked and active-unmasked conditions (551.33 ± 182.98 versus 657.67 ± 231, p > 0.05). A repeated measures ANOVA also revealed significant difference between the CEA in the early time window (CEA _(25–80 ms)_) between the three single pulse conditions (F(2, 28) = 19.96, *p < *0.001, η^2^ = 0.59) (Fig. 5a). The difference between CEA _(25–80 ms)_ active-masked and sham conditions (133.62 ± 68.15 versus 43.48 ± 20.3, *p < *0.01), and active-unmasked and sham (130.49 ± 49.63 versus 43.48 ± 20.3, *p < *0.001) were also significant while the difference between CEA _(25–80 ms)_ of two active conditions did not reach the significance level (*p* > 0.05).

#### Paired-pulse TMS (LICI)

Significant differences can be seen between the amplitude of the waveforms across three conditions in paired-pulse protocol (Fig. 2c,d). A repeated measures ANOVA revealed a significant difference in CEA _(25–275 ms)_ between the three different conditions (F(2, 28) = 10.09, p = 0.001, η^2^ = 0.42) (Fig. 3b). Post-hoc analyses further revealed a difference between CEA _(25–275 ms)_ of the active-masked and sham conditions (384.57 ± 140.54 versus 222.53 ± 132.58, *p < *0.01). A significant difference was also observed between the CEA _(25–275 ms)_ of active-unmasked and sham conditions (475.9 ± 201.96 versus 222.53 ± 132.58, *P < *0.01). However, the difference between the CEA _(25–275 ms)_ of active-masked and active-unmasked conditions did not reach significance (384.57 ± 140.54 versus 475.9 ± 201.96, P > 0.05). A repeated measures ANOVA (with Greenhouse–Geisser correction) also revealed significant difference between the CEA in the early time window (CEA _(25–80 ms)_) between three conditions (F(1.38, 19.33) = 4.25, *p < *0.05, η^2^ = 0.23) (Fig. 5b). The difference between CEA _(25–80 ms)_ active-masked and sham conditions (81.61 ± 39 versus 63.4 ± 33.19, *p < *0.001), active-unmasked and sham (118.7 ± 75.01 versus 63.4 ± 33.19, *p < *0.001), and active-masked and active-unmasked (81.61 ± 39 versus 118.7 ± 75.01, *p < *0.001) conditions were also significant.

### Global mean field amplitude (GMFA)

#### Single-pulse TMS

**Figure 4:**
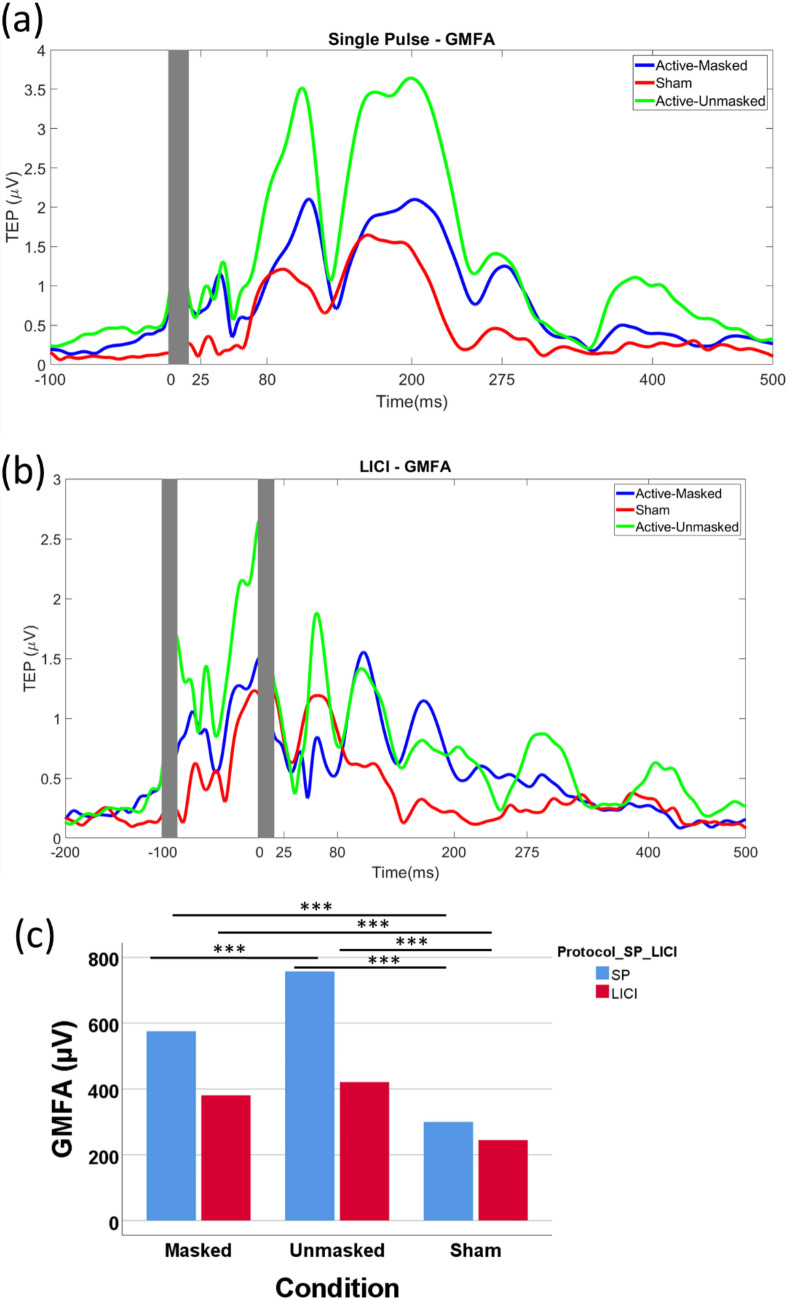
(**a**) Global mean field amplitude (GMFA) for the three single-pulse (SP) stimulation conditions. (**b**) GMFA for the three stimulation conditions of paired pulse (i.e. long interval cortical inhibition (LICI)). (**c**) Comparison of GMFA _(25–275 ms)_ for the three SP and LICI conditions using one-way repeated measure ANOVA (*** *p < *0.001).

There was a significant difference in GMFA _(25–275 ms)_ between the active-masked, active-unmasked, and sham conditions (F(2, 28) = 24.68, *p < *0.001, η^2^ = 0.64) (Fig. 4a). The difference between active-masked and sham was statistically significant (575.52 ± 189.21 versus 299.81 ± 75.66, *p* = 0.001). The difference between active-unmasked and sham was also significant (757.45 ± 237.07 versus 222.53 ± 132.58, *p < *0.001). The difference between the active-masked and active-unmasked conditions was also significant (*p < *0.001) (Fig. 4c).

**Figure 5:**
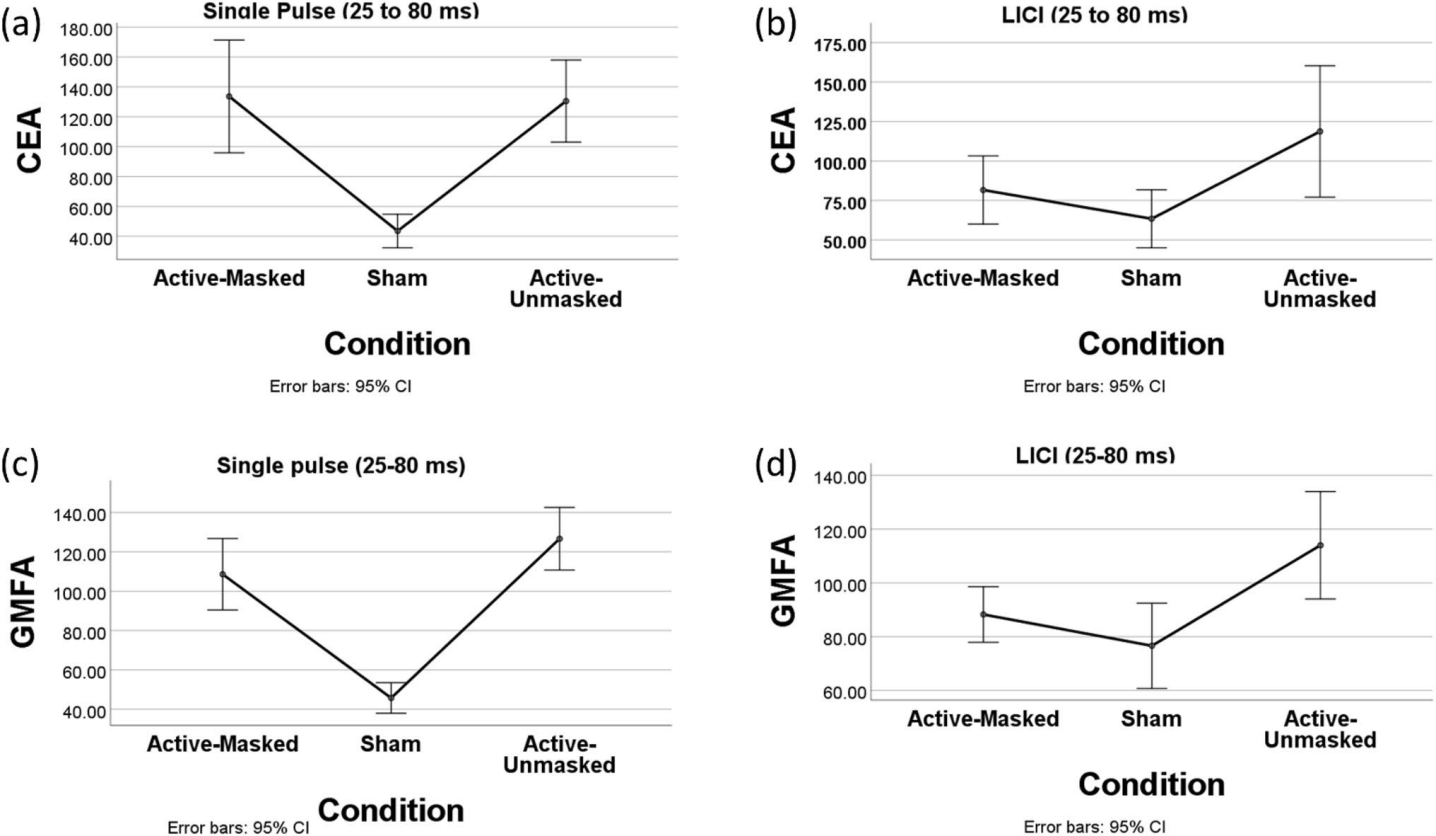
(**a**) Comparison of CEA _(25–80 ms)_ for the three single pulse conditions using one-way repeated measure ANOVA. (**b**) Comparison of CEA _(25–80 ms)_ for the three long interval cortical inhibition (LICI) conditions using one-way repeated measure ANOVA. (**c**) Comparison of GMFA _(25–80 ms)_ for the three single pulse conditions using one-way repeated measure ANOVA. (**d**) Comparison of GMFA _(25–80 ms)_ for the three LICI conditions using one-way repeated measure ANOVA. CEA is recorded from electrode F3. GMFA reflects the activity recorded across all the electrodes.

A repeated measures ANOVA also revealed significant difference between the GMFA _(25–80 ms)_ of the three single pulse conditions (F(2, 28) = 21.23, *p < *0.001, η^2^ = 0.6) (Fig. 5c). The difference between GMFA _(25–80 ms)_ active-masked and sham conditions (108.57 ± 32.79 versus 45.7 ± 13.99, *p < *0.001), and active-unmasked and sham (126.62 ± 28.74 versus 45.7 ± 13.99, *p < *0.001) were also significant while the difference between GMFA _(25–80 ms)_ of two active conditions did not reach the significant level (*p* > 0.05).

#### Paired-pulse TMS (LICI)

For the LICI protocol, a statistically significant difference was observed between the three stimulation conditions (F(2, 28) = 14.29*, p < *0.001, η^2^ = 0.5) (Fig. 4b). Pairwise comparisons revealed significant differences between GMFA _(25–275 ms)_ for the active-masked and sham conditions (380.81 ± 93.11 versus 244.71 ± 73.53, *p* = 0.001), as well as the active-unmasked and sham conditions (420.9 ± 126.25 versus 244.71 ± 73.53, *p* = 0.001) (Fig. 4c). No statistically significant difference was observed between two active LICI conditions (*p* > 0.05). A repeated measures ANOVA also revealed significant a difference between the GMFA in the early time window (GMFA _(25–80 ms)_) between the three conditions (F(2, 28) = 9.19, *p < *0.01, η^2^ = 0.4) (Fig. 5d). The difference between GMFA _(25–80 ms)_ active-unmasked and sham conditions (113.99.3 versus 76.59 ± 7.39, *p < *0.01), and active-unmasked and active-masked (113.99.3 versus 88.25 ± 4.82, *p < *0.05) was also significant, while the difference between GMFA _(25–80 ms)_ of active masked and sham did not reach the significance level (*p* > 0.05).

### TEP components

#### Components measured from GMFA

**Figure 6:**
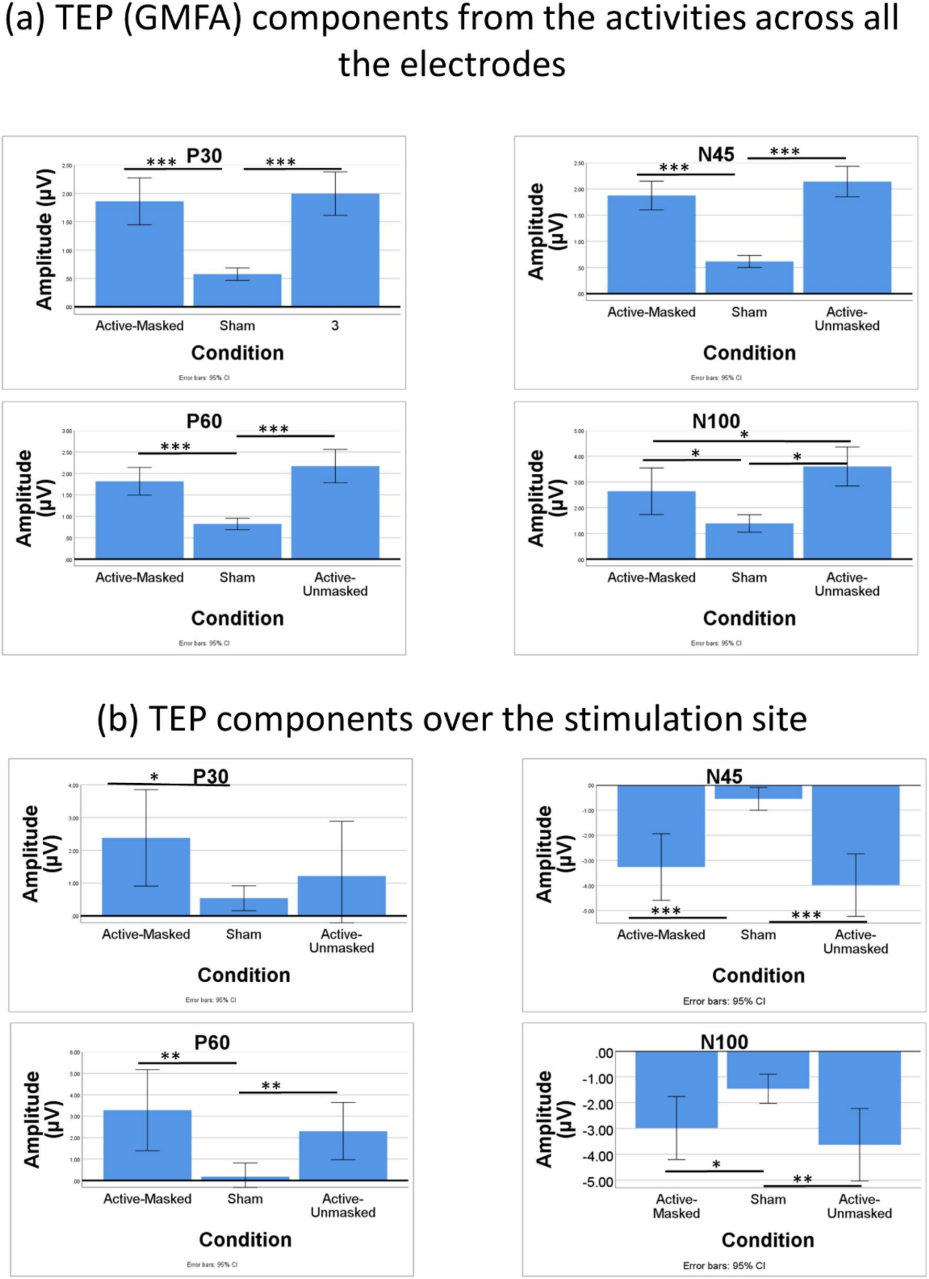
(**a**) TEP (GMFA) components from the activities across all the electrodes (**b**) TEP components over the stimulation site (* *p < *0.05, ** *p < *0.01, *** *p < *0.001).

A repeated measures ANOVA revealed that P30_(GMFA)_ differed significantly between the three conditions following single pulse TMS (F(2, 28) = 42.7*, p < *0.001, η^2^ = 0.75). Post-hoc analysis (with Bonferroni correction) revealed a significant difference between P30_(GMFA)_ for the active-masked and sham conditions (1.86 ± 0.75 versus 0.58 ± 0.2, *p < *0.001), as well as between the active-unmasked and sham conditions (1.97 ± 0.69 versus 0.58 ± 0.2, *P < *0.001). However, no significant difference was observed between the P30_(GMFA)_ of active-masked and active-unmasked conditions (1.86 ± 0.75 versus 1.97 ± 0.69, p > 0.05) (Fig. 6a).

A repeated measures ANOVA revealed that N45_(GMFA)_ differed significantly between the three conditions following single-pulse TMS (F(2, 28) = 97.88, *p < *0.001, η^2^ = 0.87). Post-hoc analysis (with Bonferroni correction) revealed a significant difference between N45_(GMFA)_ for the active-masked and sham conditions (1.88 ± 0.49 versus 0.61 ± 0.21, *p < *0.001), as well as between the active-unmasked and sham conditions (2.14 ± 0.53 versus 0.61 ± 0.21, *P < *0.001). However, no significant difference was observed between the N45_(GMFA)_ of active-masked and active-unmasked conditions (1.88 ± 0.49 versus 2.14 ± 0.53, *p* > 0.05) (Fig. 6a).

A repeated measures ANOVA revealed that P60_(GMFA)_ differed significantly between the three conditions following single pulse TMS (F(2, 28) = 35.26, *p < *0.001, η^2^ = 0.72). Post-hoc analysis (with Bonferroni correction) revealed a significant difference between P60_(GMFA)_ for the active-masked and sham conditions (1.81 ± 0.58 versus 0.82 ± 0.24, *p < *0.001), as well as between the active-unmasked and sham conditions (2.17 ± 0.7 versus 0.82 ± 0.24, *p < *0.001). However, no significant difference was observed between the P60_(GMFA)_ of active-masked and active-unmasked conditions (1.81 ± 0.58 versus 2.17 ± 0.7, p > 0.05) (Fig. 6a).

A repeated measures ANOVA revealed that N100_(GMFA)_ differed significantly between the three conditions following single pulse TMS (F(2, 28) = 23.78, *p < *0.001, η^2^ = 0.63). Post-hoc analysis (with Bonferroni correction) revealed a significant difference between N100_(GMFA)_ for the active-masked and sham conditions (2.64 ± 1.63 versus 1.39 ± 0.61, *p < *0.05), as well as between the active-unmasked and sham conditions (3.6 ± 1.37 versus 1.39 ± 0.61, *p < *0.05). Moreover, a significant difference was observed between the N100_(GMFA)_ of active-masked and active-unmasked conditions (2.64 ± 1.63 versus 3.6 ± 1.37, *p < *0.05) (Fig. 6a).

#### Components measured from the site of the stimulation

A repeated measures ANOVA revealed that P30 did not differ significantly between the three conditions following single pulse TMS (F(2, 28) = 2.95, *p < *0.001, η^2^ = 0.17). Post-hoc analysis (with Bonferroni correction) revealed a significant difference between P30 for the active-masked and sham conditions (2.38 ± 2.66 versus 0.54 ± 0.69, *p < *0.05), but no significant difference was observed between the P30 of active-unmasked and sham (1.21 ± 3.02 versus 0.54 ± 0.69, *p* > 0.05) as well as active-masked and active-unmasked conditions (2.38 ± 2.66 versus 1.21 ± 3.02, p > 0.05) (Fig. 6b).

A repeated measures ANOVA revealed that N45 differed significantly between the three conditions following single pulse TMS (F(2, 28) = 14.04*, p < *0.001, η^2^ = 0. 5). Post-hoc analysis (with Bonferroni correction) revealed a significant difference between N45 for the active-masked and sham conditions (-3.26 ± 2.39 versus -0.55 ± 0.82, *p < *0.01), as well as between the active-unmasked and sham conditions (-3.98 ± 2.45 versus -0.55 ± 0.82, *P < *0.001). However, no significant difference was observed between the N45 of active-masked and active-unmasked conditions (-3.26 ± 2.39 versus -3.98 ± 2.45, p > 0.05) (Fig. 6b).

A repeated measures ANOVA revealed that P60 differed significantly between the three conditions following single pulse TMS (F(2, 28) = 8.57*, p < *0.01, η^2^ = 0.37). Post-hoc analysis (with Bonferroni correction) revealed a significant difference between P60 for the active-masked and sham conditions (3.28 ± 3.41 versus 0.18 ± 1.15, *p < *0.01), as well as between the active-unmasked and sham conditions (2.3 ± 2.41 versus 0.18 ± 1.15, *p < *0.01). However, no significant difference was observed between the P60 of active-masked and active-unmasked conditions (3.28 ± 3.41 versus 2.3 ± 2.41, *p* > 0.05) (Fig. 6b).

A repeated measures ANOVA revealed that N100 differed significantly between the three conditions following single pulse TMS (F(2, 28) = 4.89*, p < *0.05, η^2^ = 0.26). Post-hoc analysis (with Bonferroni correction) revealed a significant difference between N100 for the active-masked and sham conditions (− 2.99 ± 2.2 versus − 1.46 ± 1.02, *p < *0.05), as well as between the active-unmasked and sham conditions (− 3.63 ± 2.53 versus − 1.46 ± 1.02, *p < *0.01). However, no significant difference was observed between the N100 of active-masked and active-unmasked conditions (− 2.99 ± 2.2 versus − 3.63 ± 2.53, *p* > 0.05) (Fig. 6b).

### Interaction effect and LICI

A two-way repeated measures ANOVA revealed no significant interaction between the masking condition (active-masked and active-unmasked) and the time window of the recorded response over the site of stimulation (CEA _(25–80 ms)_ and CEA _(25–275 ms)_) in either single pulse (F(1, 14) = 2.65, *p* = 0.13, η^2^ = 0.16) or LICI protocols (F(1, 14) = 1.52, *p* = 0.24, η^2^ = 0.1).

A two-way repeated measures ANOVA revealed a significant interaction between the masking condition and the time window of the recorded response across all of the electrodes (GMFA _(25–80 ms)_ and GMFA _(25–275 ms)_) for the single pulse (F(1, 14) = 26.49, *p < *0.0001, η^2^ = 0.65) but not for the LICI protocol (F(1, 14) = 0.37, p = 0.55, η^2^ = 0.03).

**Figure 7:**
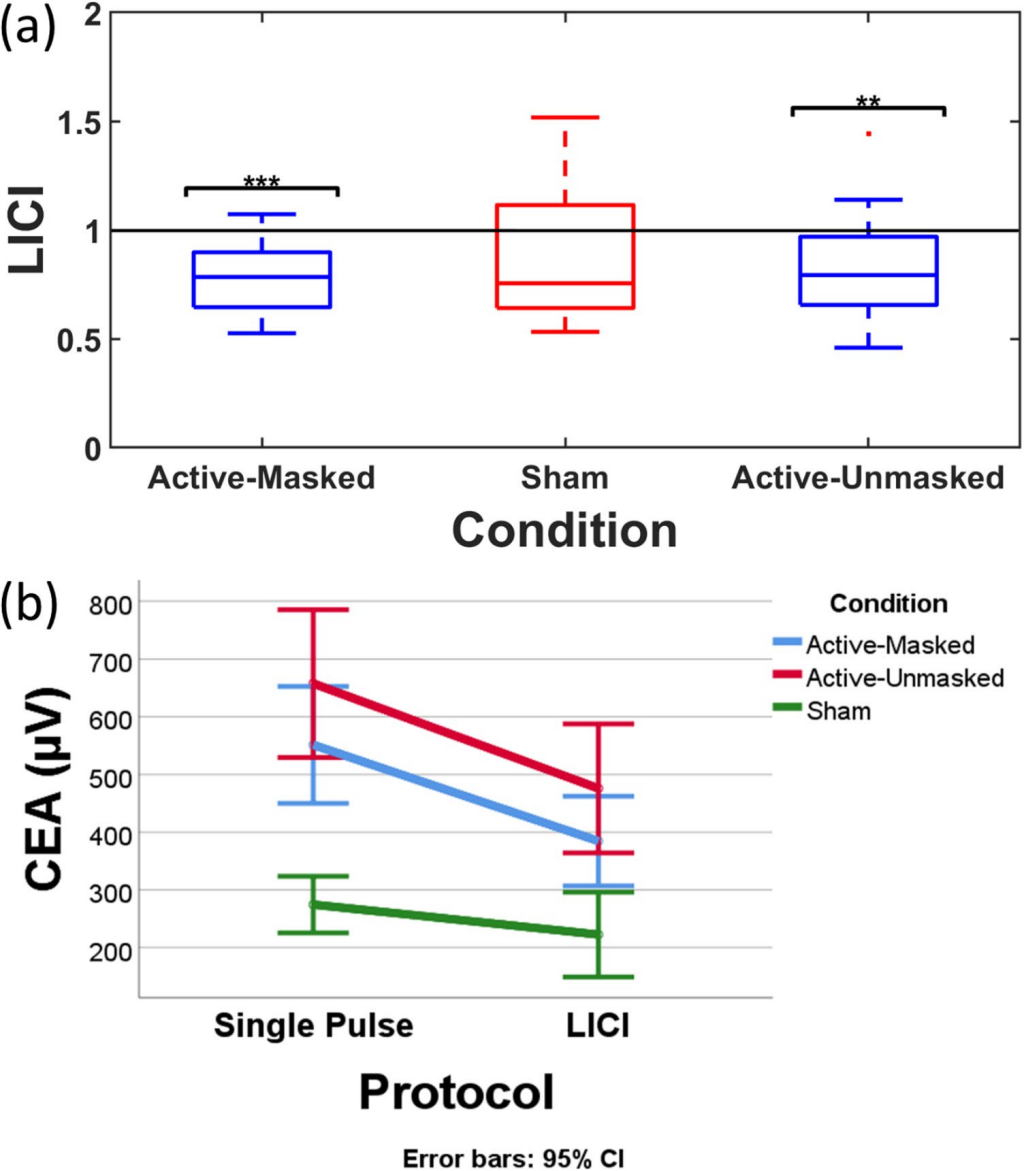
(**a**) The level of LICI in the three conditions of active-masked, active-unmasked, and sham. Only active blocks of stimulation resulted in significant signal inhibition which is shown by asterisks. (** *p < *0.01, *** *p < *0.001). (**b**) The interaction effect of condition of the stimulation and the stimulation protocol on cortical evoked activity (CEA_(25–275 ms)_) with the 95% confidence interval shown by the error bars. CEA is recorded from electrode F3.

Furthermore, a two-way repeated measures ANOVA revealed a significant main effect of stimulation condition (active-masked, active-unmasked, and sham) on CEA between active-masked and sham (*P < *0.001), and active-unmasked and sham (*p < *0.001). However, the interaction between condition and stimulation protocol of the stimulation did not reach significance (F(1.43, 20.07) = 1.74, *p* = 0.2, η^2^ = 0.11) (Fig. 7b).

Finally, the Mann–Whitney U-test revealed that active-masked (0.78 ± 0.16, z = 4.3, *p < *0.0001, r = 1.11) and active-unmasked (0.83 ± 0.25, z = 2.97, *p < *0.01, r = 0.77) LICI protocols resulted in significant LICI. Sham LICI did not reach significance (0.9 ± 0.34, z = 1.64, p = 0.1) (Fig. 7a). The comparison between LICIs in 3 conditions did not reach the significance level (F(1.29, 18.1) = 0.75, *p* = 0.43, η^2^ = 0.05).

### Single-pulse and LICI protocols: global scalp analysis

**Figure 8:**
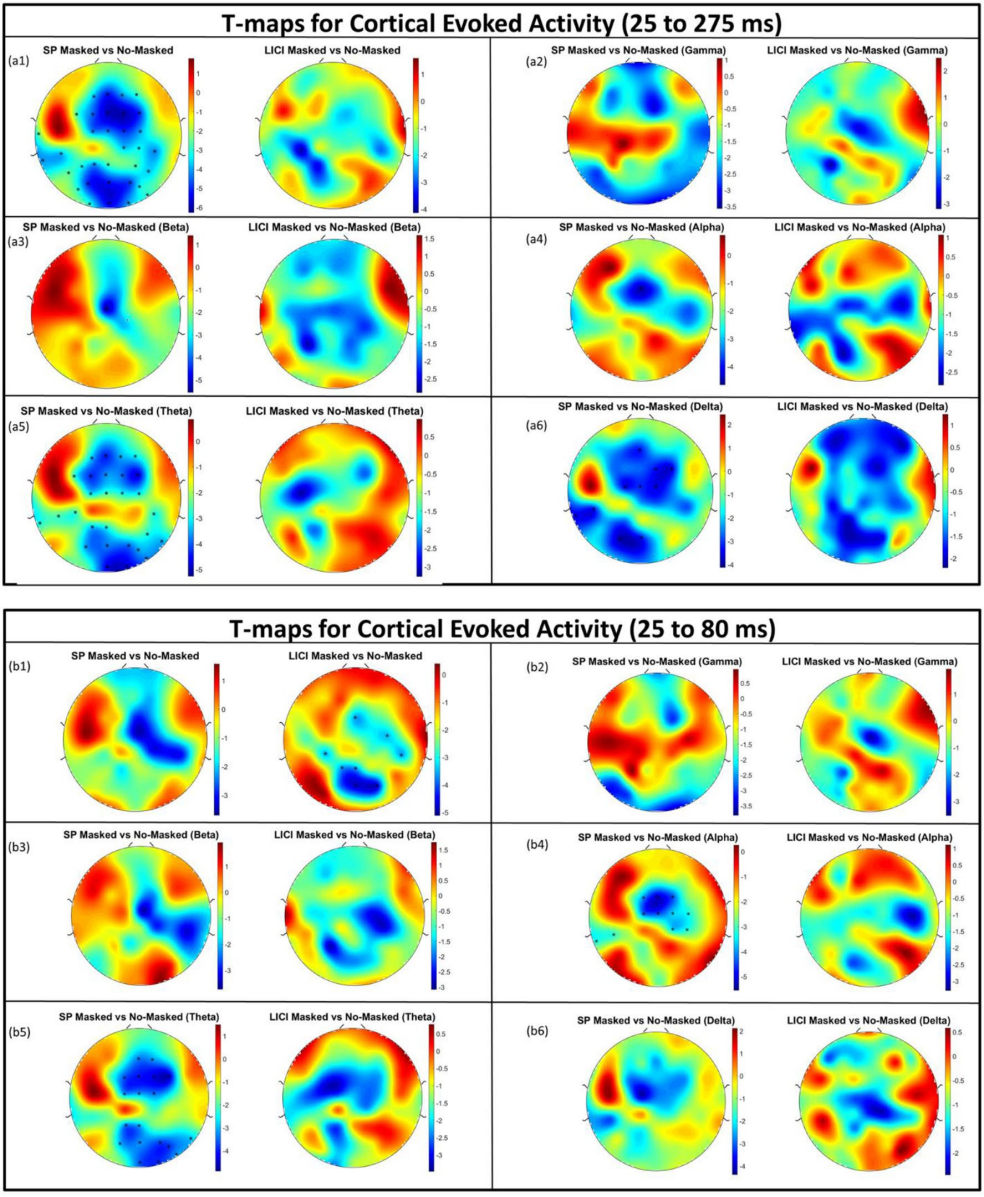
(**a1**) T-maps illustrating the t-values as the result of comparison of cortical evoked activity (CEA _(25–275 ms)_) between active-masked and active-unmasked conditions for all of the electrodes and single pulse (SP) and long interval cortical inhibition (LICI) protocols. (**a2**) T-maps illustrating the t-values as the result of comparison of CEA _(25–275 ms)_ between active-masked and active-unmasked for all of the electrodes in SP and LICI protocols over gamma band. (**a3**) T-maps illustrating the t-values as the result of comparison of CEA _(25–275 ms)_ between active-masked and active-unmasked for all of the electrodes in SP and LICI protocols over beta band. (**a4**) T-maps illustrating the t-values as the result of comparison of CEA _(25–275 ms)_ between active-masked and active-unmasked for all of the electrodes in SP and LICI protocols over alpha band. (**a5**) T-maps illustrating the t-values as the result of comparison of CEA _(25–275 ms)_ between active-masked and active-unmasked for all of the electrodes in SP and LICI protocols over theta band. (**a6**) T-maps illustrating the t-values as the result of comparison of CEA _(25–275 ms)_ between active-masked and active-unmasked for all of the electrodes in SP and LICI protocols over delta band. (**b1**) T-maps illustrating the t-values as the result of comparison of cortical evoked activity (CEA _(25–80 ms)_) between active-masked and active-unmasked conditions for all of the electrodes and single pulse (SP) and LICI protocols. (**b2**) T-maps illustrating the t-values as the result of comparison of CEA _(25–80 ms)_ between active-masked and active-unmasked for all of the electrodes in SP and LICI protocols over gamma band. (**b3**) T-maps illustrating the t-values as the result of comparison of CEA _(25–80 ms)_ between active-masked and active-unmasked for all of the electrodes in SP and LICI protocols over beta band. (**b4**) T-maps illustrating the t-values as the result of comparison of CEA _(25–80 ms)_ between active-masked and active-unmasked for all of the electrodes in SP and LICI protocols over alpha band. (**b5**) T-maps illustrating the t-values as the result of comparison of CEA _(25–80 ms)_ between active-masked and active-unmasked for all of the electrodes in SP and LICI protocols over theta band. (**b6**) T-maps illustrating the t-values as the result of comparison of CEA _(25–80 ms)_ between active-masked and active-unmasked for all of the electrodes in SP and LICI protocols over delta band. The electrodes where the corrected p-value as the result of t-test to compare between the CEA of active-masked and active-unmasked was significant are represented by an asterisk (*).

T-maps were used to investigate the difference in the signal activation topography, across all of the electrodes, between active-masked and active-unmasked conditions in both single-pulse and LICI protocols and over the canonical frequency bands. (Fig. 8). No significant difference in CEA _(25–275 ms)_ of LICI protocol was observed between the two active conditions (Fig. 8a1). Regarding the single pulse CEA _(25–275 ms)_, the posterior and central electrodes showed significant differences, but the electrodes over the site of the stimulation and contralateral to the site of the stimulation did not show significant differences. Furthermore, the frequency analysis revealed that the CEA of single-pulse and LICI active protocols did not differ significantly over any of the electrodes in gamma, beta, and alpha frequency bands (Fig. 8a2–a4). Significant differences were observed only between active protocols for single-pulse over theta and delta frequency bands but not in the LICI protocol (Fig. 8a4,a6). Moreover, the t-maps for single-pulse CEA _(25–80 ms)_ revealed no significant difference between the two active conditions with regards to the early response over any of the electrodes. For the early time window (CEA _(25–80 ms)_) of LICI protocol, the t-maps also revealed no significant difference between the active-masked and active-unmasked conditions around and contralateral to the site of the stimulation. The difference was observed only over a few posterior and central electrodes (Fig. 8b1). Finally, the frequency analysis revealed that the there was no significant difference between CEA _(25–80 ms)_ of active masked and active-unmasked conditions in single-pulse and LICI protocols over delta, beta, and gamma bands (Fig. 8b2,b3,b6). However, in theta and alpha bands, there was a significant difference between CEA _(25–80 ms)_ of single-pulse and over the central (alpha and theta) and posterior electrodes but not in LICI protocol (Fig. 8b4,b5).

### Discussion

#### Summary

The current study aimed to clarify the effect that masking the auditory and somatosensory co-activation can have on the TMS-EEG signal recorded from the left DLPFC at a suprathreshold level of stimulation by comparing two different stimulation conditions (active-masked and active-unmasked) and two protocols (single-pulse and paired pulse). We further compared the signal recoded from single-pulse and paired-pulse sham blocks of stimulation with active protocols. Our results revealed no significant difference between the response from the two active conditions over the stimulation site in both overall activity, measured by CEA _(25–275 ms)_, and CEA _(25–80 ms)_, and the TEP components (P30, N45, P60, and N100), stating the minimal contamination of the signal recorded form the site of the stimulation. Moreover, we observed no significant difference between the early responses across all electrodes (GMFA _(25–80 ms)_, P30_(GMFA)_, N45_(GMFA)_, and P60_(GMFA)_) between the two active conditions. However, both the GMFA_(25–275)_, and N100_(GMFA)_ differed significantly between the two active conditions. The similarities and differences in GMFA measures of the two active conditions emphasize the minimum effect of sensory co-activation on the signal in the early time window across all of the electrodes, and the importance of masking these sensory co-activations to achieve a less contaminated signal. As expected, we observed distinctively stronger activation in the active stimulation conditions (active-masked, active-unmasked), compared to sham, for both the single-pulse and paired-pulse (LICI) protocols, illustrating the significant difference between the level of cortical activation and sensory co-activation as the result of our sham stimulation. Finally, only active-masked and active-unmasked LICI conditions resulted in significant signal inhibition, indicating that the LICI measure is sensitive to direct TMS cortical activation rather than being elicited by indirect sensory processes.

#### Comparison between the two active conditions

The similarities and differences between the active-masked and active-unmasked conditions with regards to CEA amplitude over the site of the stimulation, as well as the topographic distribution of the signal over the scalp, provide insight about both the reliability of the TMS-EEG signal and the effect of masking sensory co-activations. We compared two active conditions—one in which no auditory or somatosensory masking was applied, and one in which the TMS pulse was delivered while white noise was played through earphones (with additional noise attenuation via earmuffs) and a layer of foam was attached to the coil to reduce sensory input. The difference between the CEA_(25–275 ms)_ in both single-pulse and LICI protocols was not significant although the CEA _(25–275 ms)_ for the stimulation without foam or auditory masking tended to be higher. More importantly, the P30, N45, P60, and N100 components did not show any significant difference between the two active conditions. Since the masking of sensory co-activations was done effectively according to the best practices in the field^12^, this finding shows the minimal effect of these sources of artifacts on the signal recorded from the electrode around the stimulation site. This is in agreement with the recent research indicating that TEPs recorded from the site of the stimulation are less contaminated by the co-activation sensory artifacts^7,9,12^.

On the other hand, the significant difference between the GMFA_(25–275 ms)_, and N100_(GMFA)_ of single-pulse active-masked and active-unmasked conditions shows that the influence of auditory evoked potentials (AEP) and somatosensory evoked potentials (SEP) on the global response is prominent if the masking is not done properly. This was confirmed by the result of our interaction analysis where the interaction effect of masking-condition and time was significant, implying that the effect that masking has on the GMFA (i.e. the global signal recorded from all the electrodes) of single-pulse stimulation depends on the time window of interest where the early time window seems free of artifact over all the electrodes but not the late response. Importantly, the non-significant difference between GMFA_(25–80 ms)_ of active-masked and active-unmasked reveals that the evoked response by TMS over all the electrodes is relatively artifact-free in the early time window. This was also confirmed by the non-significant difference between the components of the GMFA waveform in the two active conditions (P30_(GMFA)_, N45_(GMFA)_, and N100_(GMFA)_). Biabani et al.^7^ reported less contamination of the signal over the site of stimulation and in an earlier time window (prior to 100 ms post-stimulation) by comparing the TMS over the motor cortex and delivering the pulses over the shoulder (contralateral to the hemisphere of the stimulation). It has been recently shown^11,12^ that the early TEP may genuinely reflect cortical reactivity to TMS.

Examining LICI protocol signal across all the electrodes can also be informative. The difference between GMFA_(25–80 ms)_ of LICI active-masked and active-unmasked was significant. This seems to be in-line with the aforementioned significant difference between GMFA_(25–275 ms)_ of active-masked and active-unmasked single-pulse. The co-activation, introduced to the signal by the first pulse, extends into the signal recoded from the second pulse. Therefore, the early global responses of two active LICI conditions happen to be different. On the other hand, the GMFA_(25–275 ms)_ of the two active conditions in the LICI protocol do not differ significantly, which may be the result of either high level of variability in the recorded signal, or the adaptation to the auditory activation as a result of the first and second pulse (resulting in lower level of auditory evoked potential for the unmasked condition). This finding emphasizes on the importance of masking the sensory artifacts in the TMS-EEG signal.

As mentioned earlier, the TMS-EEG signal is reported to be less contaminated by the auditory and somatosensory artifacts in the early time window (before 100 ms)^7,8,11,12^. We observed no significant interaction between masking-condition and time (CEA _(25–80 ms)_, CEA _(25–275 ms)_) on the level of evoked reactivity over the site of the stimulation in neither single pulse nor LICI protocols. This finding implies that the effect of masking is not dependent on the time window analyzed (i.e., early or late response) when examining neuronal activity over the site of stimulation. Further, the T-maps revealed no significant difference between CEA_(25–275 ms)_ single-pulse active-masked and active-unmasked conditions for the electrodes around the site of stimulation and contralateral to the stimulation site. Moreover, as illustrated by the t-maps over various frequency bands, it can be seen that the difference between single-pulse active-masked and active-unmasked is reflected over the signal in theta and delta frequency bands. In other words, the AEP and SEP co-activation that is not attenuated by masking procedures and is reflected in active-unmasked condition, is reflected in the decomposed signal over theta and delta frequency bands. These observations also reaffirm the reliability of the signal recorded from the site of the stimulation. In fact, the AEP seems to be picked-up by the electrodes located over the motor cortex, while the electrodes over the frontal area and contralateral to it do not seem to be affected. Moreover, the t-maps that compared the CEA _(25–80 ms)_ of the two active conditions illustrate the reliability of early TMS-EEG signal in both single pulse and LICI protocols as there was no significant difference between the evoked activity over any of the electrodes in single-pulse and majority of the electrodes in LICI protocols.

#### LICI

Importantly, LICI seems to be robust and less affected by these two sources of artifacts. Both active-masked and active-unmasked protocols produced significant signal inhibition, while sham stimulation did not. The comparison between two active conditions indicates that the suppression of the cortical response measured by LICI was, on average, greater in active-masked compared with active-unmasked condition. Perhaps the subtraction of the artifact that coincides in the single-pulse and test pulse activations (in a similar degree) makes the LICI ratio more resilient to contamination by the auditory and somatosensory artifacts as it is shown by the current findings. The significant LICI over the DLPFC has been demonstrated in previous reports across both healthy and diseased states^16,17,23,34^. This finding is also in accordance with our previous results where the active paired-pulse protocol resulted in significant LICI, whereas auditory stimulation alone did not^10^.

#### Implications

The somatosensory and auditory co-activation of the TMS-EEG signal has been recognized as a potential source of concern since the early development of this technique. In recent years, this topic has been explored in more detail via various stimulation protocols across a number of different research groups^7–14,35^. The present study represents the first time that both similarities and differences between single-pulse and LICI protocol responses recorded with advanced methods of delivering TMS-EEG, older methods, and sensory co-activation (sham) have been directly compared using suprathreshold stimulation intensities over the prefrontal cortex. We have shown that the level of cortical reactivity, elicited by active TMS (either masked or unmasked), differs significantly from sham stimulation. We further observed that the response recorded over the site of the stimulation, as well as the early evoked response across all electrodes appear to be relatively free form sensory co-activation and reflect the cortical response. Therefore, it can be concluded that the TMS-EEG signal recorded from the prefrontal cortex at suprathreshold intensity reliably captures the cortical response to TMS activation. Finally, in order to validate that the location and intensity are sufficient to achieve effective cortical activation, we considered the peak-to-peak amplitude of the early components in our study (~ 4 µA) and validated that it is similar to the amplitude of previous publications exploring TEPs over the DLPFC^10,16,19,20,36–39^.

## Limitations

Although we have tried to follow the best practice available to apply the gold standard of TMS-EEG recording, our study has limitations. The main limitation of this study, and all the TMS-EEG studies that use sham stimulation, is how accurately the sham stimulation mimics the sensory co-activation without directly activating the cortex. We have observed significant differences in the amplitude of the sham response compared with the two active conditions (higher in active conditions) in both single-pulse and LICI protocols. This difference has been true both with regards to the activation over the site of the stimulation, illustrated by CEA, as well as the TEP components, and the topographic distribution of the signal over all the electrodes, measured by GMFA. This finding emphasizes the minimal contribution of the somatosensory and auditory activation, without activating the cortex, induced by our sham stimulation. We should emphasize that the sham coil used in our study induces a much lower level of magnetic stimulation than active stimulation. While this sham procedure has the advantage of preventing the direct activation of the cortex, it comes with the disadvantage of lower levels of sensory co-activation too. Therefore, while the lower level of response, recorded from sham stimulation compared with active, shows the minimal level of auditory and somatosensory co-activation caused by our sham control, it makes the comparison between these sensory co-activations of active and sham protocols rather difficult. This finding is in agreement with the recent findings by Rocchi et al. (2021) using TMS applied over the motor cortex at subthreshold levels (90% RMT) and using the sham coil. Gordon et al. (2018) also reported significantly lower levels of TEP amplitude with sham stimulation (electrical stimulation of the scalp) compared to suprathreshold and subthreshold stimulation intensities over motor cortex. However, in these two studies, the same concern with regards to the similarity of sensory co-stimulation between active and sham blocks seems to be valid. A lower level of activation with sham stimulation is also consistent with our previous findings ^10^ where auditory stimulation resulted in significantly lower TEP amplitudes compared to active stimulation in both single-pulse and LICI protocols over the DLPFC. On the other hand, our results are not in accordance with those of Conde et al. (2019) and Gordon et al. (2021) who demonstrated that somatosensory and auditory co-activation from sham stimulation resembled the typical TEP response, specifically in late responses. This difference may be the result of different experimental procedures (e.g. coil type and the methods used for determining the intensity of the stimulation) as well as the specific study design used (such as different types of sham stimulation and pre-processing steps) between our described study and the studies by Conde et al. (2019) and Gordon et al. (2021). Another limitation in our study was the number of participants which was limited to 15 due to the restrictions imposed as the result of COVID-19 pandemic.

Finally, it should be noted that although we have followed the best current practice of TMS-EEG, complete suppression of the somatosensory co-activation is not possible as the activation of nerve fibers can still take place.

## Conclusion

The similarities and differences observed between the level of cortical excitation and inhibition caused by various blocks of stimulation in each protocol illustrates the existence of somatosensory and auditory co-activation within the TMS-EEG signal over the prefrontal cortex. However, it can be clearly seen that the non-significant level of somatosensory contamination of the signal on one hand (recorded from sham stimulation), and the non-significant differences between neurophysiological measures of active protocols (masked and un-masked) on the other hand, make the TMS-EEG technique an effective tool to study cortical neurophysiology. Furthermore, the responses recorded over the site of the stimulation and in the early time window (25–80 ms) more reliably reflect responses from cortical activation rather than the contaminated signal. Future studies can be focused on development of more effective offline approaches for pre-processing the data, integration of machine learning algorithms for distinguishing between signal and noise, more realistic sham procedures that will not introduce unnecessary contamination of the signal, and the application of computational models to differentiate the cortical responses from the somatosensory and auditory co-activations.

## Data Availability

The datasets used during the current study are available by contacting Dr. Daniel Blumberger (daniel.blumberger@camh.ca) on reasonable request.

## Abbreviations

TMS: Transcranial magnetic stimulation
EEG: Electroencephalography
TEP: TMS evoked potential
GMFA: Global mean field potential
AEP: Auditory evoked potential
SEP: Somatosensory evoked potential
SP: Single pulse
PP: Paired pulse
LICI: Long-interval intracortical inhibition;
AUC: Area under the curve
CEA: Cortical evoked activity
DLPFC: Dorsolateral prefrontal cortex
MRI: Magnetic resonance imaging
MNI: Montreal neurological institute
APB: Abductor pollicis brevis
ICA: Independent component analysis
